# Comparison of the efficacy of aroma-acupressure and aromatherapy for the treatment of dementia-associated agitation

**DOI:** 10.1186/s12906-015-0612-9

**Published:** 2015-03-29

**Authors:** Man-Hua Yang, Li-Chan Lin, Shiao-Chi Wu, Jen-Hwey Chiu, Pei-Ning Wang, Jaung-Geng Lin

**Affiliations:** Department of Nursing, National Yang-Ming University, 155, Li-Nong Street Sec 2, Beitou District Taipei, 11221 Taiwan; Institute of Clinical and Community Health Nursing, National Yang-Ming University, 155, Li-Nong Street Sec 2, Beitou District Taipei, 11221 Taiwan; Institute of Health and Welfare Policy, National Yang-Ming University, 155, Li-Nong Street Sec 2, Beitou District Taipei, 11221 Taiwan; Institute of Traditional Medicine, National Yang-Ming University, 155, Li-Nong Street Sec 2, Beitou District Taipei, 11221 Taiwan; Faculty of Medicine, National Yang-Ming University, 155, Li-Nong Street Sec 2, Beitou District Taipei, 11221 Taiwan; Institute of Traditional Chinese Medicine, China Medical University, 91, Hsueh-Shuh Road, Taichung, 40402 Taiwan

**Keywords:** Dementia, Agitation, Aroma-acupressure, Aromatherapy

## Abstract

**Background:**

One of the most common symptoms observed in patients with dementia is agitation, and several non-pharmacological treatments have been used to control this symptom. However, because of limitations in research design, the benefit of non-pharmacological treatments has only been demonstrated in certain cases. The purpose of this study was to compare aroma-acupressure and aromatherapy with respect to their effects on agitation in patients with dementia.

**Methods:**

In this experimental study, the participants were randomly assigned to three groups: 56 patients were included in the aroma-acupressure group, 73 patients in the aromatherapy group, and 57 patients in the control group who received daily routine as usual without intervention. The Cohen-Mansfield Agitation Inventory (CMAI) scale and the heart rate variability (HRV) index were used to assess differences in agitation. The CMAI was used in the pre-test, post-test and post-three-week test, and the HRV was used in the pre-test, the post-test and the post-three-week test as well as every week during the four-week interventions.

**Results:**

The CMAI scores were significantly lower in the aroma-acupressure and aromatherapy groups compared with the control group in the post-test and post-three-week assessments. Sympathetic nervous activity was significantly lower in the fourth week in the aroma-acupressure group and in the second week in the aromatherapy group, whereas parasympathetic nervous activity increased from the second week to the fourth week in the aroma-acupressure group and in the fourth week in the aromatherapy group.

**Conclusions:**

Aroma-acupressure had a greater effect than aromatherapy on agitation in patients with dementia. However, agitation was improved in both of the groups, which allowed the patients with dementia to become more relaxed. Future studies should continue to assess the benefits of aroma-acupressure and aromatherapy for the treatment of agitation in dementia patients.

**Trial registration:**

ChiCTR-TRC-14004810; Date of registration: 2014/6/12

## Background

Agitation is one of the most commonly observed symptoms in dementia patients. Agitation includes inappropriate physical and verbal actions that cause trouble for family members and caregivers and can even lead to lost work and other financial burdens. Antipsychotics have generally been effective for the treatment of psychosis and agitation in patients with dementia. However, the number of side effects associated with these drugs has increased, and cerebrovascular adverse events, including stroke, have been noted in elderly patients with dementia taking first-generation or second-generation antipsychotic drugs [1]. Compared with pharmacological treatments, non-pharmacological treatments, such as acupressure and aromatherapy, have ameliorated agitation and cognitive impairment in dementia patients [2-4]. Non-pharmacological treatments are non-invasive, have fewer side effects and are safer to use [5]. Although medical scientists from many backgrounds have used acupressure in their studies to alleviate symptoms such as agitation and sleeplessness [6,7], these studies did not measure the physiological parameters associated with agitation, which weakened their evidence in support of non-pharmacological treatment effects. However, according to some research, sleeplessness and agitation are related to the level of sympathetic nervous activity [8]. When the sympathetic nervous system is hyperactive, resulting in an increase in plasma catecholamine levels, lower cognitive ability, and higher cognitive fatigue, several consequences occur including high blood pressure, agitation, and arrhythmia [9], as well as the potential inhibition of parasympathetic nervous activity. In contrast, when the parasympathetic nervous system is activated, blood pressure and heart rate decrease. Most previous studies have been limited by small sample sizes. For example, the systematic review by Lee, Shin, and Ernst was unable to measure the effect of acupuncture treatment on Alzheimer’s disease (AD) because of the small sample size associated with randomized controlled trials (RCTs) [10]. Therefore, future clinical trials should follow standard procedures and include sufficient sample sizes.

Aromatherapy has been widely applied to treat agitation in dementia patients. The absorption of essential oil via transdermal administration or inhalation may activate the autonomic nervous system and induce the reaction of the limbic system and hypothalamus [11]. Thus, this treatment would facilitate feelings of relaxation and decrease agitation in patients with dementia. Lavender and lemon balms are two common essential oils that are used in aromatherapy, and studies have shown that these plants possess calming and relaxing qualities, promote healthy sleep [12,13], ameliorate agitation, and improve quality of life in dementia patients. However, similar to previous studies on acupressure, these experimental studies included small samples and few objective physiological parameters, which led to difficulties in interpretation of the results [14]. Therefore, the purpose of this study was to perform a rigorous clinical trial to explore the potential ability of aroma-acupressure and aromatherapy to improve agitation in dementia patients, with the aim of generating a simple and nonintrusive protocol that could be provided to caregivers.

## Methods

### Participants

For this experimental study, participants were recruited from 6 institutions that specialize in the care of dementia patients in Taiwan. These institutions included three retirement homes for veterans and three long-term care facilities. We generated the allocation sequence based on the properties of the institutions that used stratified sampling to put the six institutions into two categories, i.e. veteran home and long term care facility and adopted the principle of equal allocation to encode the two categories respectively. Each time, an institution in the veteran home was randomly assigned to the aroma-acupressure, aromatherapy, or control group and so was an institution in the long term care facility. The research assistant was blinded to the assignment procedure and allocation results. The data were collected from February 1, 2012 to May 31, 2013, and the following inclusion criteria were used: (i) fulfilled the DSM-IV standard for dementia as diagnosed by psychiatrists or neurologists; (ii) scored 35 or above on the long form of the Cohen-Mansfield Agitation Inventory (CMAI), which was defined as severe agitation; (iii) expected to be present in the long-term care facility every Monday to Friday during the period of the study; and (iv) did not possess broken skin or infection surrounding the acupoints.

According to the G-power 3.0 analysis, a total sample size of 108 is required when α = .05, and the effect size is.25, which would yield a power of.80 with three groups and three repeated measurements.

This study was approved by a full board review of the Taipei City Hospital Institutional Review Board (IRB) (No. TCHIRB-1000912). During recruitment, the study goals and procedures were explained to the heads of the institutions and the family members of the participants before the informed consent forms were signed.

### Interventions

The Baihui (GV 20), Fengchi (GB 20), Shenmen (HT 7), Neiguan (PC 6), and Sanyinjiao (SP 6),acupuncture points were used in the aroma-acupressure protocols to treat agitation [15]. The operation time in each protocol consisted of the following: (i) each acupoint was pressed for 2 minutes with 2.5% lavender oil and (ii) a warm-up exercise was completed for 5 minutes. The duration of each protocol was no longer than 15 minutes, and each protocol was conducted once per day for five days per week for four weeks total. For the aromatherapy group, 2.5% lavender oil was applied at five acupoints with the same operation time as the aroma-acupressure group. In the control group, the daily care routine continued as usual without interventions.

### Instruments

The long form of the CMAI, which consists of 29 types of problem behavior and provides scores that range from 1 (never occur) to 7 (occur several times per hour), was used to track the frequency of problem behavior in one week [16]. Lin has previously used the Chinese version of the CMAI and improved the scale’s reliability and validity [17]. Therefore, the Chinese version was used to measure agitation in the dementia patients.

A heart rate variability (HRV) analyzer (8Z11, Enjoy Research Inc., Taiwan) [18] was used to acquire, store, and process the electrocardiogram (ECG) signals. The low frequency percentage (LF%) and the low frequency-high frequency (LF/HF) ratio were used to represent sympathetic nervous activity, and the high frequency (HF) was used to represent parasympathetic nervous activity.

### Study procedure

In the pre-test, the research assistant was responsible for completing the CMAI and the HRV. The HRV was used to measure time in the pre-test and every week during the four-week interventions following the pre-test. Following the completion of the interventions, the data from the post-test and post-three week assessments were collected. In order to control the environmental factors that might affect HRV results, all participants were measured after lunch break in their own rooms.

### Statistical analyses

All statistical analyses were performed using the software SPSS 20.0 (IBM Corporation, Armont, NY, USA). Descriptive statistics were used to describe the characteristics of the participants. The three groups were compared using one-way ANOVA and chi-square tests. A generalized estimating equation (GEE) for repeated measurements was used to assess the outcome indicators. Based on intention-to-treat analyses, which included subjects with missing data points, we used the missing-at-random assumption to conduct the GEE analysis.

## Results

**Figure 1:**
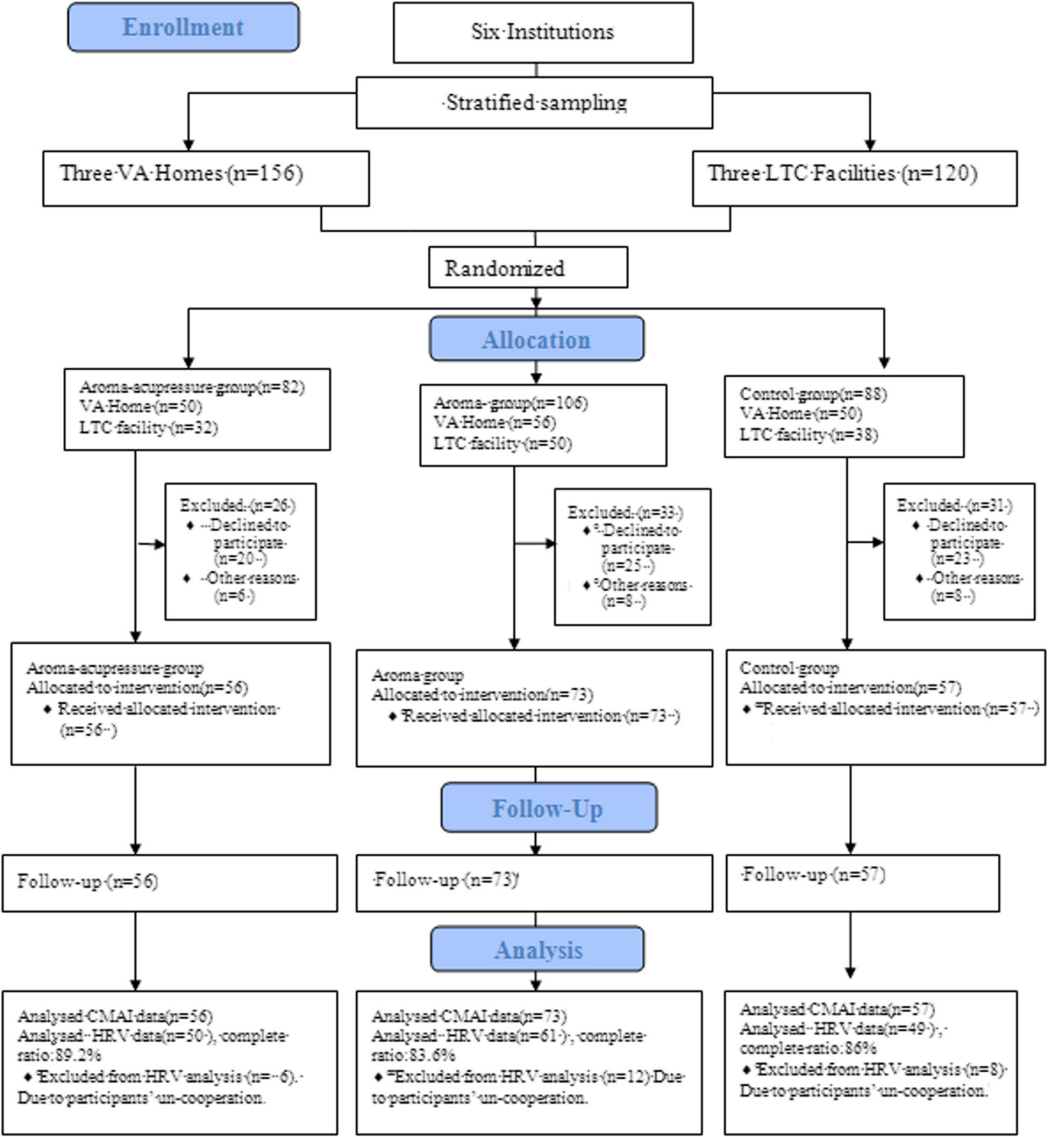
**Participant flow in the study.**

Of the 276 qualified participants, 21 participants were hospitalized, 1 participant was deceased, and 68 participants chose not to participate in the study. Thus, 186 participants were included in the study and were randomly assigned to the three groups. Figure 1 depicts the flow of the participants through each stage. The demographic data of the three groups are shown in Table 1. The average age was 85.3 years in the aroma-acupressure group, 83.67 years in the aromatherapy group, and 81.56 years in the control group, with significant age differences between the 3 groups. However, there were no significant differences among the groups in regards to sex. Regarding the types of dementia present, the majority of the participants in the three groups were AD patients. Two types of restraints were used: a physical constraint that involved the use of a restraint belt, and a chemical restraint that involved the use of antipsychotic drugs. There were no significant differences in the use of restraints among the three groups.

**Table 1:** **Demographic data**

	**Aroma-acupressure group (n = 56)**	**Aroma group (n = 73)**	**Control group (n = 57)**	**F** ^**a**^ **/χ2** ^**b**^	**P value**
**Age, mean ± SD or n (%)**	85.3 ± 5.76	83.67 ± 4.96	81.56 ± 6.79	5.91^a^ 20.83^b^	<0.01* <0.01*
**65-74 years**	4 (7.1%)	3 (4.1%)	11 (19.3%)		
**75-84 years**	14 (25%)	39 (53.4%)	25 (43.9%)		
**≧85 years**	38 (67.9%)	31 (42.5%)	21 (36.8%)		
**Sex, n (%)**				5.69^b^	0.06
**Male**	46 (82.1%)	48 (65.8%)	43 (75.4%)		
**Female**	10 (17.9%)	25 (34.2%)	14 (24.6%)		
**Diagnosis of Dementia, n (%)**				20.40^b^	<0.01*
**AD**	45 (80.3%)	70 (95.9%)	55 (96.5%)		
**Vascular**	10 (17.9%)	0 (0%)	2 (3.5%)		
**Other**	1 (1.8%)	3 (4.1%)	0 (0%)		
**Restraint, n (%)**				5.67^b^	0.23
**None**	29 (51.8%)	35 (47.9%)	31 (54.4%)		
**One (chemical/physical)**	22 (39.2%)	35 (47.9%)	25 (43.9%)		
**Both**	5 (9%)	3 (4.2%)	1 (1.7%)		

*Note.* ^a^One-way ANOVA; ^b^Chi-square test; *p < 0.05.

### Outcome and estimation

The comparisons of the CMAI among the groups are demonstrated in Table 2. In both the aroma-acupressure and aromatherapy groups in the pre- and post-tests, the CMAI scores were significantly higher compared with the control group. Over time, the scores in the aroma-acupressure group significantly declined, with the post-test score lower compared with the post-three week score. A similar decline was also observed in the aromatherapy group, although there was little difference between the two scoring times. Accordingly, both the aroma-acupressure and aromatherapy groups experienced improvement in agitation.

**Table 2:** **Changes in the CMAI scores over time for the three groups**

	**Pre-test Mean ± SD**	**Post-test Mean ± SD**	**Post −3 weeks test Mean ± SD**	**β (95%CI)**	**p value**
**Group**					
A-a group (n = 56)	54.58 ± 11.01	43.24 ± 10.00	51.21 ± 11.95	16.74(13.71—19.77)	0.00*
A group (n = 73)	41.81 ± 7.89	41.08 ± 8.24	39.80 ± 7.27	4.01(1.19—6.83)	0.01*
Control group (n = 57)	37.68 ± 4.12	41.72 ± 5.08	42.13 ± 5.53	reference	
**Time**					
**Post-test**				3.96(2.22-5.71)	<0.01*
**Post- 3-week**				4.39(2.64-6.13)	<0.01*
**Group x Time**					
**A-a group x post-test**				−15.31(−17.83 to −12.79)	<0.01*
**A-a group x post-3 weeks**				−7.65(−10.20 to −5.11)	<0.01*
**A group x post-test**				−4.82(−7.19 to −2.45)	<0.01*
**A group x post-3 weeks**				−5.93(−8.31 to −3.56)	<0.01*

*Note.*; Aroma-acupressure group: A-a group; Aroma group: A group; CI: confidence interval; * p < 0.05.

Regarding the results of the HRV test (see Table 3) for the sympathetic nervous system, the LF/HF in the aroma-acupressure group was significantly higher compared with the control group. Over time, the LF% and LF/HF in the aroma-acupressure group did not exhibit a weekly difference. However, a group interaction with time was present, which was demonstrated by the weekly decline in LF% every week until the fourth week. A similar result occurred in the aromatherapy group, and there was a significant difference in the second week. In addition, the weekly LF/HF was significantly reduced each week until the fourth week; however, this declining trend was not present in the aromatherapy group.

**Table 3:** **Changes in the LF%, LF/HF, and HF over time in the three groups**

	**LF%**		**LF/HF**		**HF**	
	**β (95%CI)**	**p**	**β (95%CI)**	**p**	**β (95%CI)**	**p**
**Group**						
**A-a group (n = 50)**	7.86 (−0.23-15.95)	0.06	0.79 (0.04-1.54)	0.04*	−0.22 (−1.31-0.87)	0.69
**A group (n = 61)**	0.25 (−7.00-7.49)	0.95	0.43 (−0.25-1.11)	0.22	0.35 (−0.68-1.37)	0.51
**Control group (Reference, n = 49)**						
**Time**						
**1** ^**st**^ **week**	−3.29 (−6.69-3.12)	0.32	−0.01 (−0.69-0.66)	0.98	−0.20 (−1.12-0.73)	0.68
**2** ^**nd**^ **week**	1.94 (−4.51-8.39)	0.56	0.21 (−0.47-0.89)	0.55	−0.16 (−1.10-0.78)	0.74
**3** ^**rd**^ **week**	−5.68 (−12.63-1.27)	0.11	−0.06 (−0.79-0.68)	0.87	0.06 (−0.95-1.06)	0.91
**4** ^**th**^ **week**	−1.06 (−7.70-5.58)	0.75	0.06 (−0.64-0.76)	0.87	−0.36 (−1.31-0.60)	0.47
**Post-test**	−1.73 (−8.86-5.39)	0.63	0.05 (−0.70-0.80)	0.90	−0.07 (−1.09-0.96)	0.90
**Post-3 wks**	−0.77 (−7.50-5.96)	0.82	0.11 (−0.60-0.82)	0.76	−0.20 (−1.17-0.77)	0.69
**Group x Time**						
**A-a group x 1** ^**st**^ **week**	−1.51 (−11.05-8.20)	0.76	−0.45 (−1.44-0.54)	0.37	1.00 (−0.33-2.34)	0.14
**A-a group x 2** ^**nd**^ **week**	−8.93 (−18.37-0.51)	0.06	−0.82 (−1.80-0.16)	0.10	2.91 (1.60-4.23)	<0.01*
**A-a group x 3** ^**rd**^ **week**	−9.38 (−19.35-0.60)	0.07	−0.91 (−1.94-0.12)	0.08	8.54 (7.17-9.92)	<0.01*
**A-a group x 4** ^**th**^ **week**	−12.34 (−22.16 to −2.5)	0.01*	−1.09 (−2.11 to −0.07)	0.04*	5.45 (4.08-6.82)	<0.01*
**A-a group x post-test**	−9.90 (−20.15-0.34)	0.06	−0.83 (−1.89-0.23)	0.13	0.68 (−0.76-2.11)	0.36
**A-a group x post-3 wks**	−7.16 (−17.27-2.96)	0.17	−0.50 (−1.55-0.55)	0.35	0.54 (−0.87-1.96)	0.45
**A group x 1** ^**st**^ **week**	−5.39 (−13.87-3.91)	0.21	−0.50 (−1.39-0.40)	0.28	−0.43 (−1.65-0.80)	0.49
**A group x 2** ^**nd**^ **week**	−12.09 (−20.6 to −3.59)	0.01*	−0.15 (−1.05-0.75)	0.75	0.27 (−0.96-1.50)	0.67
**A group x 3** ^**rd**^ **week**	0.39 (−8.57-9.35)	0.93	−0.47 (−1.41-0.48)	0.33	−0.42 (−1.65-0.80)	0.52
**A group x 4** ^**th**^ **week**	−5.74 (−14.43-2.95)	0.20	−0.71 (−1.63-0.20)	0.13	1.58 (0.32-2.83)	0.01*
**A group x post-test**	−8.98 (−18.33-0.36)	0.06	−0.69 (−1.67-0.29)	0.17	0.88 (−0.47-2.22)	0.20
**A group x post-3 wks**	−2.09 (−11.32-7.14)	0.57	−0.48 (−1.46-0.49)	0.33	0.45 (−0.89-1.78)	0.51

*Note.* Aroma-acupressure group: A-a group; Aroma group: A group; CI: confidence interval; *p < 0.05.

For the parasympathetic nervous system, there were no significant differences between the three groups or the assessment times. In the group interaction with time, the HF in the aroma-acupressure group was significantly higher in the second, third, and fourth weeks, whereas the HF was significantly higher only in the fourth week for the aromatherapy group. The effects of aroma-acupressure on the parasympathetic nervous system were also stronger over time.

The participants did not experience side effects at any stage of the interventions.

## Discussion

The CMAI score was significantly higher in the aroma-acupressure and aromatherapy groups compared with the control group in the pre- and post-tests; the average scores in the pre- and post-tests were highest in the aroma-acupressure group, followed by the aromatherapy group and the control group. Prior to the interventions, agitation was significantly more severe in the aroma-acupressure and aromatherapy groups compared with the control group, and the CMAI score was significantly higher in the post-test and post-three-week assessments compared with the pre-test. In the aroma-acupressure and aromatherapy groups, the pre-test CMAI score was significantly higher compared with the post-test and post-three-week scores, which indicates that both aroma-acupressure and aromatherapy can immediately and persistently improve agitation. This finding is consistent with previous studies demonstrating that both aroma-acupressure and aromatherapy can improve agitation [6,7]. These effects may persist for three weeks following the interventions. After further comparison of the declining CMAI scores between the aroma-acupressure and aromatherapy groups in the post-test and post-three-week assessments, the decline in the aroma-acupressure group was larger compared with that in the aromatherapy group, which demonstrates that aroma-acupressure was better able to improve agitation compared with aromatherapy.

According to the HRV, the LF/HF was significantly higher compared with the control group only in the aroma-acupressure group; each measurement in the aroma-acupressure group was significantly higher compared with the control group. The result is generally consistent with previous acupuncture study, which showed the sympathetic nervous system activity in the aroma-acupressure group was stronger compared with the control group [19]; this finding is also consistent with the agitation results previously described. Regarding the group interaction with time, the sympathetic nervous system (LF% and LF/HF) in the aroma-acupressure group did not demonstrate a significant decline until the fourth week following the interventions. This result indicates that the relaxing effect of aroma-acupressure was only present following an accumulation of time. However, only LF% in the aromatherapy group was significantly lower in the second week following the interventions, but LF/HF did not have any significant change during the period of study; this finding suggests that aroma-acupressure is better at inhibiting the sympathetic nervous system compared with aromatherapy. Both acupressure and acupuncture have the same mechanism. Acupuncture can adjust dopamine that has to do with sympathetic nervous system. Mainly, the GABA nerves of ventral tegmental area (VTA) can be activated then cause the release of dopamine from nucleus accumbens (NAc) to be inhibited [20]. As a result, in the study of Yoon et al., acupuncture on Shenmen point in order to activate the GABA_B_ receptor of the GABA nerves of VTA, thus reduce the release of dopamine from NAc distinctly, and inhibit sympathetic nervous system eventually [21].

The effect of aromatherapy is to absorb the essential oil into the circulatory system through skin. The effect mainly takes place from nerve conduction. First, the peripheral nerves are stimulated and next somatic nerves and autonomic nerves. Stimulation of somatic nerves can cause skeletal muscles to relax and stimulation of sympathetic nervous system and parasympathetic nervous system can cause blood vessels, internal organs, and glands to effect [11]. For example, Hongratanaworakit explored the relaxing effect of rose essential oil on humans when it was absorbed through skin. It was discovered that the oil could significantly lowered respiratory frequency, oxygen saturation, and systolic pressure, indicating that it could inhibit sympathetic nervous system [22]. In other words, effects of both acupuncture and aromatherapy are related to autonomic nervous system and the effect of aroma-acupressure is superior to aromatherapy, which indicates the combination effect of aromatherapy and acupressure is larger than the use of aromatherapy alone.

In the aroma-acupressure group, the parasympathetic activity (HF) was significantly higher from the second week to the fourth week following the interventions, which demonstrates the relaxing effect of aroma-acupressure; however, in the aromatherapy group, HF was significantly higher only in the fourth week following the interventions. Therefore, the effects of aroma-acupressure on enhancing the parasympathetic nervous system appear to be stronger compared with aromatherapy.

According to the HRV and the CMAI, aroma-acupressure was better able to improve agitation, inhibit the sympathetic nervous system, and activate the parasympathetic nervous system compared with aromatherapy. One explanation for this finding is that the participants in this study were primarily AD patients. According to previous studies, AD is often accompanied by olfactory impairment, and more serious AD is associated with the loss of additional olfactory abilities [23]. As a result, the ability to absorb essential oils may become weaker because essential oils are absorbed through transdermal administration or inhalation. Similarly, Snow, Hovanec, and Brandt found no support for the use of a purely olfactory form of aromatherapy to decrease agitation in dementia because olfactory impairment may weaken the effects of aromatherapy [24].

Figure 1 shows a skew distribution of participants among three groups that were 56, 73, and 57, indicating that the aromatherapy group had much more participants than other two groups. This study used stratified sampling to enroll the participants, and divided institutions into veteran’s homes and long-term care facilities. The number of residents with dementia at three veteran’s homes was 50, 56 and 50, respectively, and the number was very close. Other three long term care facilities belonged to large-scale institutions with more than 300 residents. After the randomization, this study found that the number of residents with dementia at the three facilities was 32, 50, and 38, respectively, and the number was different. The number of participants randomized to aromatherapy group was the largest, so the number of participants receiving aromatherapy was greater than that of other two groups.

The epidemiology of dementia suggests that there are more female compared with male AD patients [25]. However, this study recruited a greater number of male AD patients because three of the six chosen institutions for participant recruitment were retirement homes for veterans. Although the demographic data showed no significant difference in sex among the three groups, future studies should adopt additional long-term care facilities to recruit equal numbers of male and female participants. Another limitation was the experimental study design, as the participants were aware of the specific group they were assigned to. In future studies, participants should be treated with base oil or pressed at false acupoints to ensure a double-blind study design.

## Conclusions

This experimental study confirms the beneficial effects of non-pharmacological treatments on agitation in patients with dementia. In both the aroma-acupressure and aromatherapy groups, the CMAI scores were significantly lower over time. Aroma-acupressure was better able to inhibit the sympathetic nervous system and increase the activity of the parasympathetic nervous system compared with aromatherapy. Our findings support the efficacy of non-pharmacological treatments in decreasing agitation. These non-pharmacological protocols should be more fully explored and refined in future studies.
